# Helicopter Parenting and Youth Affective Well-Being: Need Satisfaction as a Within-Family Mediator

**DOI:** 10.1007/s10964-025-02164-1

**Published:** 2025-03-18

**Authors:** Yue Wang, Savannah Boele, Anne Bülow, Loes Keijsers, Skyler T. Hawk

**Affiliations:** 1 Department of Educational Psychology, Faculty of Education, The Chinese University of Hong Kong, Hong Kong, China; 2 Department of Psychology, Education and Child Studies, Erasmus University Rotterdam, Rotterdam, Netherlands

**Keywords:** Helicopter parenting, Affective well-being, Self-determination theory, Dynamic structural equation modeling, Within-family

## Abstract

Parenting processes occur within families and unfold over time. According to Self-Determination Theory (SDT), helicopter parenting can threaten youth’s psychological need satisfaction and undermine well-being. This study represents the first investigation of these theorized within-family, time-lagged processes. The research followed 350 late adolescents in Hong Kong (*M*_age_ = 18.2, *SD*_age_ = 1.09, 39.7% male, 60.3% female, 98.9% Chinese) for an academic year, collecting 16 bi-weekly reports of maternal helicopter parenting, youth affective well-being, and youth psychological need satisfaction. Preregistered Dynamic Structural Equation Models showed that, within families, helicopter parenting predicted decreased autonomy and relatedness (but not competence) satisfaction, which subsequently predicted decreased positive affect and increased negative affect. Parenting effects were time-dependent, exhibiting differences in valence and statistical significance between concurrent and time-lagged associations. This meso-longitudinal study highlights the applicability of SDT to parenting contexts and underscores the significance of considering the timeframe in understanding parenting processes.

## Introduction

Parenting is a dynamic process that necessitates adjustments according to children’s developmental progress and changing needs (Kobak et al., 2017; Sameroff, 2010). During the transition to adulthood, parents should adapt their parenting practices to foster their children’s autonomy and self-reliance in the face of significant changes and challenges (Arnett, 2007). Parents who are not fully capable of such adaptation might resort to helicopter parenting, which is characterized by developmentally inappropriate over-involvement (Padilla-Walker & Nelson, 2012; Segrin et al., 2013). Based on Self-Determination Theory (SDT; Ryan & Deci, 2017), helicopter parents’ excessive and unnecessary involvement could thwart youth’s basic psychological needs for autonomy, relatedness, and competence, and therefore diminish their psychosocial growth and well-being (Soenens et al., 2017). Many prior studies have examined these theorized processes, mostly using between-family designs (i.e., trait-like differences between families; see review: Cui et al., 2022) or approaches that confound between- and within-family processes (e.g., Gao et al., 2022), but none to date have investigated how these processes operate within families (i.e., state-like information depicting changes between a particular adolescent and his/her parent over time). As parenting processes naturally occur within a family unit, disentangling underlying dynamics within families from stable between-family differences is necessary to advance theory and practice (Hamaker, 2012; Keijsers, 2016). The present study therefore examined within-family associations between helicopter parenting, youth psychological need satisfaction, and their affective well-being.

**Fig. 1:**
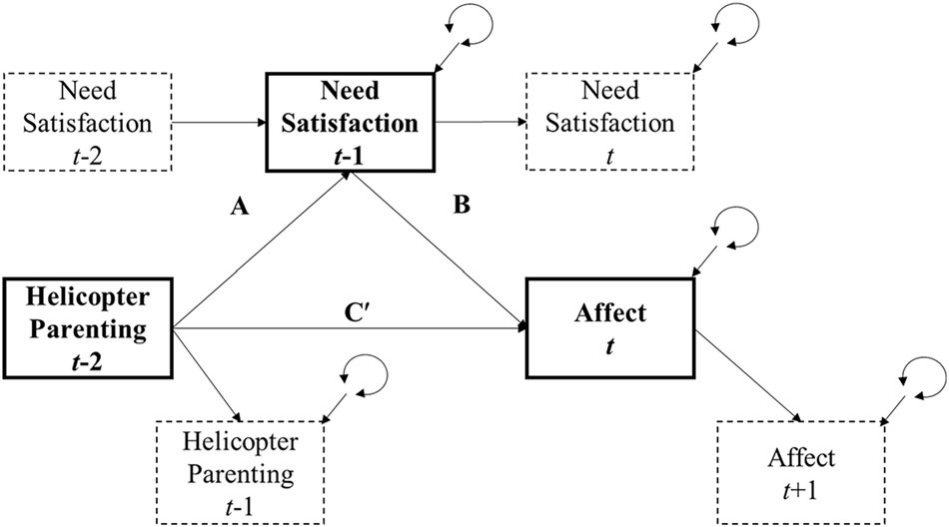
The Theoretical Model for the Within-Family Associations between Study Variables. *Note*. The time unit is two weeks. Rectangles represent observed variables. The residual variances of the study variables are person-specific. Path A refers to helicopter parenting predicting need satisfaction (autonomy, relatedness, and competence satisfaction) one time unit later. Path B refers to need satisfaction predicting youth affect (positive affect and negative affect in separate models) one time unit later. Path C’ refers to helicopter parenting predicting youth affect two time units later. All paths are modeled as being constant across both time and individuals. The figure is adapted from McNeish & MacKinnon (2022). This figure only displays the within-person processes tested in the model; the full path diagram associated with this model can be found at https://osf.io/qkza9/

The use of analytical approaches that confound between-family and within-family processes in previous longitudinal studies also complicates investigation of the temporal sequence of proposed relations between helicopter parenting, autonomy satisfaction, and emotional difficulties (e.g., Gao et al., 2022). The underlying mediating processes, from helicopter parenting to need satisfaction and subsequent impacts on changes in well-being, have never been assessed at the appropriate conceptual level of inference. To address these limitations, this study utilized advanced mediation models within the framework of Dynamic Structural Equation Modeling (DSEM; Asparouhov et al., 2018; McNeish & MacKinnon, 2022) to investigate the within-family, time-lagged links between maternal helicopter parenting, youth psychological need satisfaction (i.e., autonomy, relatedness, and competence satisfaction), and affective well-being (i.e., positive and negative affect; See Fig. 1).

### Links between Helicopter Parenting and Adolescents’ Well-Being

Theories on helicopter parenting suggest that it can diminish youth well-being through misalignments between parents’ involvement and their children’s developmental needs (Padilla-Walker & Nelson, 2012; Segrin et al., 2013). While helicopter parents’ excessive involvement might be well-intentioned and provide temporary benefits (e.g., for career development and learning; Cui et al., 2022), research conducted mostly in Western contexts consistently warns about the detrimental impacts of helicopter parenting on youth behavioral, social, and emotional adjustment (see reviews: Cui et al., 2022; De Roo et al., 2022). Understanding whether helicopter parenting holds consistently negative implications for youth adjustment necessitates examinations within cultural contexts such as China, where high levels of involvement are more normative (Leung & Busiol, 2016).

Parenting can be understood in terms of stable differences between families (i.e., trait-like) and dynamic processes within families (i.e., state-like; Boele et al., 2020; Keijsers et al., 2022). Most previous studies on helicopter parenting have used cross-sectional designs focused on the between-family process, which can address questions such as whether adolescents who experience higher levels of helicopter parenting, compared to others, also report lower well-being than others (see meta-analyses by De Roo et al., 2022; Zhang & Ji, 2023). In contrast, state-of-the-art analytical approaches allow for the examination of processes that occur at the within-family level, or changes over time within one family, where parenting processes actually happen (Hamaker, 2012; Keijsers, 2016). Investigating developmental perspectives on parenting dynamics within families offers a better match with developmental theories, and helps to avoid inaccurate conclusions (cf. a Simpson’s Paradox, or differently-valenced associations at between-family and within-family levels; see Nelemans et al., 2020 for an example).

Existing literature includes only three studies that have assessed helicopter parenting within families. One study among Dutch adolescents linked helicopter parenting with other parenting practices (i.e., psychological control; Luijk et al., 2023). The other two studies, conducted with Chinese families, explored its association with youth academic adjustment (Wang et al., 2023) and youth-mother relationship quality (Wang & Hawk, 2023). The only study that explored lagged effects, drawing upon the same sample as the present study but with different measures (i.e., mothers’ general perceptions of their helicopter parenting; Odenweller et al., 2014) and time intervals (three months), did not observe within-family, time-lagged effects from helicopter parenting to late adolescents’ reports of their educational identity development (Wang et al., 2023). It remains unclear whether these findings can be generalized to other aspects of well-being (e.g., affective well-being) and different timescales (e.g., several weeks).

In response to the call for increased investigations of meso-timescales in parenting research (i.e., measurement intervals of weeks or months; see Boele et al., 2020 for relevant discussions), the current study employed a bi-weekly design. By utilizing this approach, the present research can potentially contribute to the establishment of parenting theories on the meso-timescales. Though direct evidence is lacking, a recent bi-weekly study conducted with Dutch adolescents (Boele et al., 2024) provides some support for the claim that helicopter parenting could diminish adolescents’ well-being within families. Results showed detrimental impacts of psychological control on adolescents’ self-esteem and depressive symptoms over two weeks (Boele et al., 2024). Considering that helicopter parenting and psychological control share overlapping characteristics (Luijk et al., 2023), it is possible that experiencing higher levels of helicopter parenting than usual might generally precede youth’s lower affective well-being (e.g., less positive affect and more negative affect) in the following weeks.

### Basic Psychological Need Satisfaction as Within-Family Mediator

Scholars have drawn upon SDT (Ryan & Deci, 2000) to explain why helicopter parenting might be detrimental to youth well-being (e.g., Hong & Cui, 2023; Schiffrin et al., 2019). SDT represents a broad framework for studying how personal and contextual factors influence individuals’ intrinsic motivation, psychological development, and well-being (Ryan & Deci, 2000). SDT posits autonomy (i.e., experiencing a sense of self-determination and violation), relatedness (i.e., experiencing a sense of closeness and connection with others), and competence (i.e., experiencing a sense of efficacy and capability in achieving desired outcomes) as three basic psychological needs that serve as essential and universal nutrients to healthy psychological growth (Ryan, 1995; Ryan & Deci, 2000). The failure to satisfy these psychological needs predicts youth psychosocial adjustment difficulties (Ryan & Deci, 2000, 2017). Helicopter parents’ excessive involvement might undermine children’s autonomy satisfaction by limiting their ownership of goals, convey a lack of confidence in their abilities in a way that threatens their competence satisfaction (Hong & Cui, 2020; Van Petegem et al., 2020), and/or impede relatedness satisfaction because youth might feel less connected to parents due to a perceived lack of parental trust (Soenens et al., 2017). Thus, helicopter parenting might predict youth’s decreased affective well-being through reduced satisfaction of basic psychological needs.

Though the mediating role of psychological need satisfaction between helicopter parenting and youth well-being has been assessed in previous work, several theoretical considerations remain unclear. Inherently, these mediation processes imply a temporal sequence. In other words, these processes unfold over time in a manner that cannot be captured in previous cross-sectional studies (see reviews: Cui et al., 2022; De Roo et al., 2022). To date, the only study that examined the time-lagged mediating effects of autonomy satisfaction between helicopter parenting and emotional problems in Chinese adolescents (Gao et al., 2022) was restricted by confounding parenting effects at the between- and within-family levels. Since youth interact with their own mothers within the family unit, it is important to examine dynamic mediation processes with theorized time lags at the within-family level. In sum, SDT would consider helicopter parenting as a need-thwarting practice that subsequently contributes to diminished youth well-being. The current study is the first to examine the underlying time-lagged processes within families (c.f., Gao et al., 2022; Hong & Cui, 2023), which can clarify the SDT account of helicopter parenting effects in the family context and offer effective suggestions for practitioners.

## The Current Study

Empirical evidence on the theorized temporal sequence among helicopter parenting, youth’s basic psychological needs, and youth adjustment has not yet been established within families. This preregistered study examined the mediating role of adolescents’ psychological need satisfaction (at *t* -1) between helicopter parenting (at *t* - 2) and adolescent affect (at *t*) within Chinese families. A meso-longitudinal design with bi-weekly assessments followed late adolescents for a full academic year, to evaluate the applicability of SDT in explaining the time-lagged links between parental practices and youth well-being within families. First, two models (the total effects) examined the within-family associations between maternal helicopter parenting and adolescents’ positive and negative affect. It was expected that, on average, increases in helicopter parenting would be followed by decreases in positive affect (Hypothesis 1a) and increases in negative affect (Hypothesis 1b) after four weeks (i.e., two time units). Second, adolescents’ psychological need satisfaction was examined as a mediator of the associations between helicopter parenting and adolescents’ affect. For each specific need satisfaction (i.e., autonomy, relatedness, and competence satisfaction), it was expected that helicopter parenting would negatively predict adolescents’ psychological need satisfaction (Hypothesis 2a; see Fig. 1 Path A). It was further hypothesized that higher levels of adolescents’ psychological need satisfaction would predict their later reports of more positive affect (Hypothesis 2c; see Fig. 1 Path B) and less negative affect (Hypothesis 2 d; see Fig. 1 Path B). It was expected that helicopter parenting would indirectly predict adolescents’ decreased positive (Hypothesis 2 g) and increased negative affect (Hypothesis 2 h) four weeks later, through lower need satisfaction (Path A → Path B in Fig. 1). No a priori hypotheses were made regarding whether mediation processes would differ between the three psychological needs. Moreover, three sets of exploratory analyses were conducted to examine the hypothesized processes using varying timeframes, specific dimensions of helicopter parenting, and mothers’ reports of helicopter parenting behaviors.

## Method

### Participants

A total of 354 first-year college students in Hong Kong and their mothers participated in a project called “Competitive Advantages in a Threatening World”. Four dyads and one mother formally withdrew during data collection. The current study therefore included 350 adolescents (39.7% male, *M*_T1age_ = 18.20, *SD* = 1.09, Range_T1age_ = 17–24), with 90% of adolescents aged between 17 and 19. Most of these adolescents were Chinese (98.9%); all of them lived with their families throughout data collection due to social distancing regulations in Hong Kong during the COVID-19 pandemic. Adolescents reported diverse study majors, with 17.1% in arts, 12.6% in business, 9.7% in engineering, 20.6% in medicine, 11.5% in science, 16.0% in social science, and 12.6% in other fields.

Most participating mothers (*N* = 349, *M*_T1age_ = 49.10, *SD* = 4.82) were married (82.3%), some were divorced (14.6%) or widowed (2.3%), and a few were never married (0.9%). Mothers’ highest education level was primary school (12.3%), secondary school or high school (61.7%), high-diploma or associate degree (7.7%), and university degree (11.7%). Most mothers had full-time jobs (49.7%), some had part-time jobs (12.9%) or were homemakers (32.6%), and a few were unemployed (4.9%). In comparison to the median monthly household income of HK$27,500 (USD 3,483) in the year 2020, when the data collection began, the participants’ level of family monthly income was relatively low. Specifically, 23.4% of families earned less than HK$15,000 (US$1,900), 28.9% between HK$15,001 and HK$25,000 (US$1,900 to US$3,200), 28.9% between HK$25,001 and HK$45,000 (US$3,201 to US$5,700), and 28.9% higher than HK$45,001 (US$5,700).

### Procedure

Recruitment for the study was conducted between August and September 2020 among first-year college students and their mothers at a large public university in Hong Kong. The study received ethical approval from the institutional review board at the corresponding author’s university (SBRE-18-366). Multiple recruitment strategies were employed, including presentations at first-year orientation activities, as well as advertisements on the university’s social media, websites, and mass mail systems. Before participating in the study, both the adolescents and their mothers provided informed consent.

Over the course of a full academic year (from September 2020 to April 2021), adolescents and their mothers received a total of 16 bi-weekly questionnaires administered through ExpiWell, a mobile phone-based survey app. Each survey administration was accompanied by a one-week window in which participants were expected to complete the measurements. The survey took approximately five minutes to complete. Furthermore, participants filled out a baseline questionnaire (ca. 10 to 15 extra minutes) and some additional measures at the beginning and end of each semester (plus 15 minutes). Participants received a payment of HK$20 (US$2.55) per completed bi-weekly questionnaire, HK$50 (US$6.37) per comprehensive questionnaire, and HK$100 (US$12.75) bonus upon project completion.

### Missing Data

On average, adolescents completed 15.3 of the 16 bi-weekly questionnaires (95.6%). Most of the adolescents (95.1%, *n* = 333) completed at least 11 of the 16 bi-weekly questionnaires, and 81.4% (*n* = 285) completed all 16 questionnaires. Across measurement occasions, compliance ranged from 91.4% to 100%, with 94.0% at the last measurement. The missing data were completely at random (MCAR), as indicated by Little’ MCAR test (χ^2^(15708) = 13978.30, χ^2^/*df* = 0.89, *p* = 1.00). All available data were used for the analyses. The number of observations was 5,357 for each youth-reported variable.

### Measures

Measures that lacked an established Chinese version were translated and back-translated by bilingual speakers. The complete set of items used in the study can be found in Section 1 of the supplementary materials. For detailed results of psychometric properties, see Tables S1–S3 in Section 2 of the supplementary materials.

#### Helicopter parenting behavior

To assess maternal helicopter parenting behaviors, an adapted version of the Chinese Helicopter Parenting Scale (Zong & Hawk, 2022) was administered to adolescents and mothers on a bi-weekly basis. The original 16-item questionnaire is a concise, multidimensional measurement that has been validated with both Chinese adolescents and their mothers (Zong & Hawk, 2022). The original scale was modified to enhance its applicability for assessing mothers’ concrete behaviors in the past week. This involved merging similar items and excluding those that might not be appropriate for short-term measurement. Consequently, the final scale comprises 11 items that assess four distinct dimensions of helicopter parenting behaviors. Examples of adolescent-reported items include: How often has your mother… (1) “Made suggestions to help you get things accomplished” (Advice/Affect Management (AA); four item), (2) “Tried to solve a problem for you before you even experienced it” (Anticipatory Problem Solving (AP); two items), (3) “Used social media to follow your day-to-day activities” (Information Seeking (IS); three items), and (4) “Paid strong attention to your school assignments and/or exams” (Emphasis on Academic Performance (EA); two items). Items were scored from 1 (*Not at all*) to 5 (*Very often*). The same items were adapted for mothers’ reports. This shortened scale had good reliability for adolescents’ and mothers’ reports at both between-family and within-family levels for the overall score (adolescents: ω_Between_ = 0.88, ω_Within_ = 0.77; mothers: ω_Between_ = 0.92, ω_Within_ = 0.79) and for each subscale (adolescents: AA: ω_Between_ = 0.86, ω_Within_ = 0.71; AP: *r*_Between_ = 0.99, *r*_Within_ = 0.66; IS: ω_Between_ = 0.85, ω_Within_ = 0.67; EA: *r*_Between_ = 0.88, *r*_Within_ = 0.48; mothers: AA: ω_Between_ = 0.89, ω_Within_ = 0.71; AP: *r*_Between_ = 0.95, *r*_Within_ = 0.60; IS: ω_Between_ = 0.87, ω_Within_ = 0.65; EA: *r*_Between_ = 0.89, *r*_Within_ = 0.54). Multilevel Confirmatory Factor Analysis (MCFA; Geldhof et al., 2014) revealed a good fit for the four-factor model using adolescents’ reports (CFI = 0.94, TLI = 0.91, RMESA = 0.04) and mothers’ reports (CFI = 0.96, TLI = 0.94, RMESA = 0.03).

#### Psychological need satisfaction

Youth reported their experience of psychological need satisfaction and frustration through a shortened version of the Basic Psychological Need Satisfaction and Frustration Scale (BPNSFS; Chen et al., 2015). The original BPNSFS used 24 items to assess individuals’ satisfaction and frustration with their needs for autonomy, relatedness, and competence. In the current study, the BPNSFS was adapted by using six satisfaction items (Autonomy Satisfaction, e.g., “I feel a sense of choice and freedom in the things I undertake.”; Relatedness Satisfaction, e.g., “I feel that the people I care about also care about me.”; Competence Satisfaction, e.g., “I feel confident that I can do things well.”) and three frustration items (one for each need; e.g., “I feel insecure about my abilities.” in Autonomy Frustration). Adolescents answered each item on a 6-point scale (1 = *Very untrue of me*; 6 = *Very true of me*). The satisfaction items had acceptable reliability for each specific need (Autonomy satisfaction: *r*_Between_ = 0.76, *r*_Within_ = 0.35; Relatedness satisfaction: *r*_Between_ = 0.89, *r*_Within_ = 0.40; Competence satisfaction: *r*_Between_ = 0.96, *r*_Within_ = 0.61; *p*s < 0.001). Results of the MCFA for the three-factor need satisfaction model showed a good fit to the data (CFI = 0.99, TLI = 0.97, RMSEA = 0.03). Results using each specific need frustration were not explored due to the potentially uncertain reliability and limited representativeness of single items (Allen et al., 2022). Results based on combining need satisfaction and frustration (i.e., two satisfaction items and one frustration item for each specific need) were not reported due to low reliability (e.g., ω_Within_ = 0.27 for autonomy need).

#### Affective well-being

Youth’ positive and negative affect were measured at bi-weekly intervals with a self-report of the adapted Positive and Negative Affect Scale (PANAS; Hamid & Cheng, 1996; Watson et al., 1988). Positive affect includes Happy, Relaxed, Energetic, Cheerful, and Motivated (5 items); Negative affect consists of Sad, Afraid, Hostile, Anxious, Short-Tempered, and Depressed (6 items). The validation process of this adapted scale can be found at https://osf.io/qkza9/. Participants answered how often in the past week they felt these emotions on a 5-point scale from 1 (*Not at all*) to 5 (*Very often*). The internal consistency of the scale was good at the between-family level and sufficient at the within-family level (Positive affect: ω_Between =_ 0.95, ω_Within_ = 0.75; Negative affect: ω_Between_ = 0.95, ω_Within_ = 0.73). Moreover, MCFA indicated an acceptable model fit for a two-factor model (CFI = 0.91, TLI = 0.88, RMSEA = 0.04).

### Preregistered Analysis Plan

To estimate the time-lagged mediating roles of psychological need satisfaction in the associations between helicopter parenting and adolescent affective well-being, the study employed intensive longitudinal mediation models based on Dynamic Structural Equation Modeling (allowing for multivariate model; Asparouhov et al., 2018; McNeish & MacKinnon, 2022), which combines time-series analysis (enabling the modeling of lagged relations and carryover effects between repeated measures), multilevel modeling (allowing the disentanglement of stable between-family differences and over-time within-family effects), and structural equation modeling (allowing multivariate models). Given the number of time points and sample size available, stationary mediation models were used. This approach shares similarities with cross-lagged panel modeling, but offers the added advantages of being applicable to larger numbers of time points and distinguishing between-family and within-family parameters (Asparouhov et al., 2018; McNeish & MacKinnon, 2022). The hypotheses and analytical approach were preregistered (https://osf.io/qkza9/), following a similar approach in previous studies (Boele et al., 2024; Bülow, Van Roekel, et al., 2022).

All hypotheses were initially tested using adolescents’ reports. First, the mean-level structure of the data was checked. Because measurement occasion explained less than 0.3% of the variance in study variables, the mean stationarity was assumed. Eighteen participants did not show variance on at least one scale (*n* = 2 helicopter parenting, *n* = 1 positive affect, *n* = 1 negative affect, *n* = 4 autonomy satisfaction, *n* = 7 relatedness satisfaction, *n* = 11 competence satisfaction). These participants were excluded from the analysis, resulting in varying sample sizes for each model (*n*s = 336 to 347; see Table S4 in supplemental materials). Then, eight models (see Table S4 for tested paths) were fitted in M*plus* 8.3 (Muthén & Muthén, 1998–2019) with Bayesian.

To account for unequal time intervals between observations due to missing data (Hamaker et al., 2018), the TINERVAL was set to 1 (representing two weeks). Prior distributions were set to the M*plus* defaults. The model was run with a minimum of 5,000 iterations and thinning of 2. The convergence was checked by inspecting the Potential Scale Reduction (PSR) factor and trace plots. As suggested, a stable PSR lower than 1.1 or 1.05 (Asparouhov & Muthén, 2010), and a trace plot resembling a fat caterpillar (Hamaker et al., 2018) indicate adequate convergence. No convergence problems emerged. To assess the robustness of these findings, the minimal iterations were doubled to 10,000 to verify whether the PSR remained close to one and whether the results were consistent – which was the case. The reported results were based on 10,000 iterations.

In the first two models (see Table S4), the hypothesized total effects (Hypothesis 1a-b) were derived from fixed within-family lagged effects from helicopter parenting (*t* – 2) to adolescents’ positive and negative affect (*t*), respectively. In the Models 3–8 (see Table S4; two models for each specific dimension of need satisfaction), hypothesized effects were derived from fixed within-family lagged effects from helicopter parenting (*t* – 1) to psychological need satisfaction (*t*; Hypothesis 2a; *a*-path), from psychological need satisfaction (*t* – 1) to positive and negative affect (*t*; Hypothesis 2c-d; *b*-path). Finally, the model constraint statements were used to create new parameters for testing indirect (or mediation) effects (Hypothesis 2g-h; *a*-path times *b*-path). All hypotheses were supported if the 95% credible intervals (CI) of the unstandardized effects did not include zero.

The main analyses differ from the preregistration in two ways. First, indirect paths through each specific need satisfaction were examined instead of overall psychological need satisfaction, as doing so can reveal more nuanced indirect processes and potential differential effects that might be obscured by the overall construct. Second, due to the low measurement reliability of the need frustration subscale, analysis of need frustration are not presented (Hypothesis 2b, e-f, and i-j). Results of the analysis on overall need satisfaction and need frustration are available online for interested readers https://osf.io/qkza9/.

### Exploratory Analysis

Three sets of exploratory analyses were executed: 1) tests of varying timescales; 2) tests of specific helicopter parenting dimensions; and 3) tests of mother-reported helicopter parenting. The first set of exploratory analyses was also preregistered. To date, there remains a dearth of empirical studies or theoretical models explicating the timescale(s) at which psychological need satisfaction might mediate the associations between parenting behaviors and youth well-being. Therefore, as a first exploration of the underlying timescale of these parenting dynamics (see Boele et al., 2023), additional analyses were conducted to examine the proposed mediation model with varying temporal orders of study variables. Specifically, two different temporal orders were tested: (a) helicopter parenting (*t* - 1) was correlated with adolescents’ psychological need satisfaction (*t* – 1), and adolescent’s need satisfaction predicted adolescents’ affect two weeks later (*t*), and (b) a concurrent mediation model with associations between the variables examined at the same time point (*t*).

Second, exploratory models tested whether different dimensions of helicopter parenting (i.e., Advice/Affect Management, Anticipatory Problem Solving, Information Seeking, and Emphasis on Academic Performance) had varying effects on the satisfaction of each specific psychological need. Finally, additional models examined whether mother-reported helicopter parenting predicted need satisfaction and affect in ways that were consistent with youth-reported helicopter parenting.

## Results

### Descriptive Statistics and Correlations

Descriptive statistics and correlations are presented in Table 1. The ICCs indicated that 54% to 69% of the variances in the bi-weekly measures were due to stable between-family variance. The remaining 31% to 46% was due to overtime within-family changes. Youth-reported helicopter parenting was positively correlated with positive affect (*r*_w_ = 0.14, *r*_b_ = 0.24) and negative affect (*r*_w_ = 0.17, *r*_b_ = 0.26) at both levels. Additionally, helicopter parenting was positively correlated with relatedness (*r*_w_ = 0.06) and competence satisfaction (*r*_w_ = 0.04) at the within-family level, but not with autonomy satisfaction. Helicopter parenting was not correlated with any specific dimension of need satisfaction at the between-family level. Mother-reported helicopter parenting was positively correlated with youth-reported helicopter parenting (*r*_w_ = 0.12, *r*_b_ = 0.38). It was not related to positive affect and was positively related to negative affect (*r*_w_ = 0.05, *r*_b_ = 0.13) at both levels. Moreover, mother-reported helicopter parenting was positively related to autonomy (*r*_w_ = 0.04) and relatedness satisfaction (*r*_w_ = 0.03) at the within-family level. Positive affect was positively correlated with psychological need satisfaction (0.20 ≤ *r*_w_ ≤ 0.28, 0.48 ≤ *r*_b_ ≤ 0.62) at both levels. Negative affect was negatively correlated with psychological need satisfaction (−0.20 ≤ *r*_w_ ≤ −0.13, −0.47 ≤ *r*_b_ ≤ −0.51) at both levels. Positive and negative affect were negatively correlated at both levels (*r*_w_ = −0.26, *r*_b_ = −0.46).

**Table 1:** Descriptive Statistics and Within- and Between-Family Correlations (N = 350)

Variables							
	1.	2.	3.	4.	5.	6.	7.
**Youth-report**							
Helicopter Parenting	–	0.14***	0.17***	0.02	0.06***	0.04^**^	0.12***
Positive Affect	0.24***	–	−0.26***	0.20***	0.23***	0.28***	0.01
Negative Affect	0.26***	−0.46***	–	−0.16***	−0.13***	−0.20***	0.05***
Autonomy Satisfaction	−0.05	0.51***	−0.47***	–	0.38***	0.37***	0.04^**^
Relatedness Satisfaction	−0.09	0.48***	−0.51***	0.66***	–	0.36***	0.03^*^
Competence Satisfaction	0.07	0.62***	−0.51***	0.73***	0.54***	–	0.03
**Mother-report**							
Helicopter Parenting	0.38***	0.08	0.13^*^	−0.02	−0.09	0.01	–
*M*	2.36	2.95	2.30	4.00	4.22	3.99	2.45
*SD*	0.66	0.67	0.76	0.78	0.81	0.86	0.63
ICC	0.69	0.56	0.66	0.54	0.56	0.58	0.65

*N* = 350 for youth-reported variables, *n* = 349 for mother-reported helicopter parenting. Correlations above the diagonal line represent within-family correlations and below the diagonal line represent between-family correlations. *M* = mean. *SD* = standard deviation. ICC = intraclass correlation. *N* = sample size

^*^p < 0.05, ^**^p < 0.01, ***p < 0.001

### Total Effects of Helicopter Parenting on Adolescents’ Affect (*Hypothesis 1*)

**Fig. 2:**
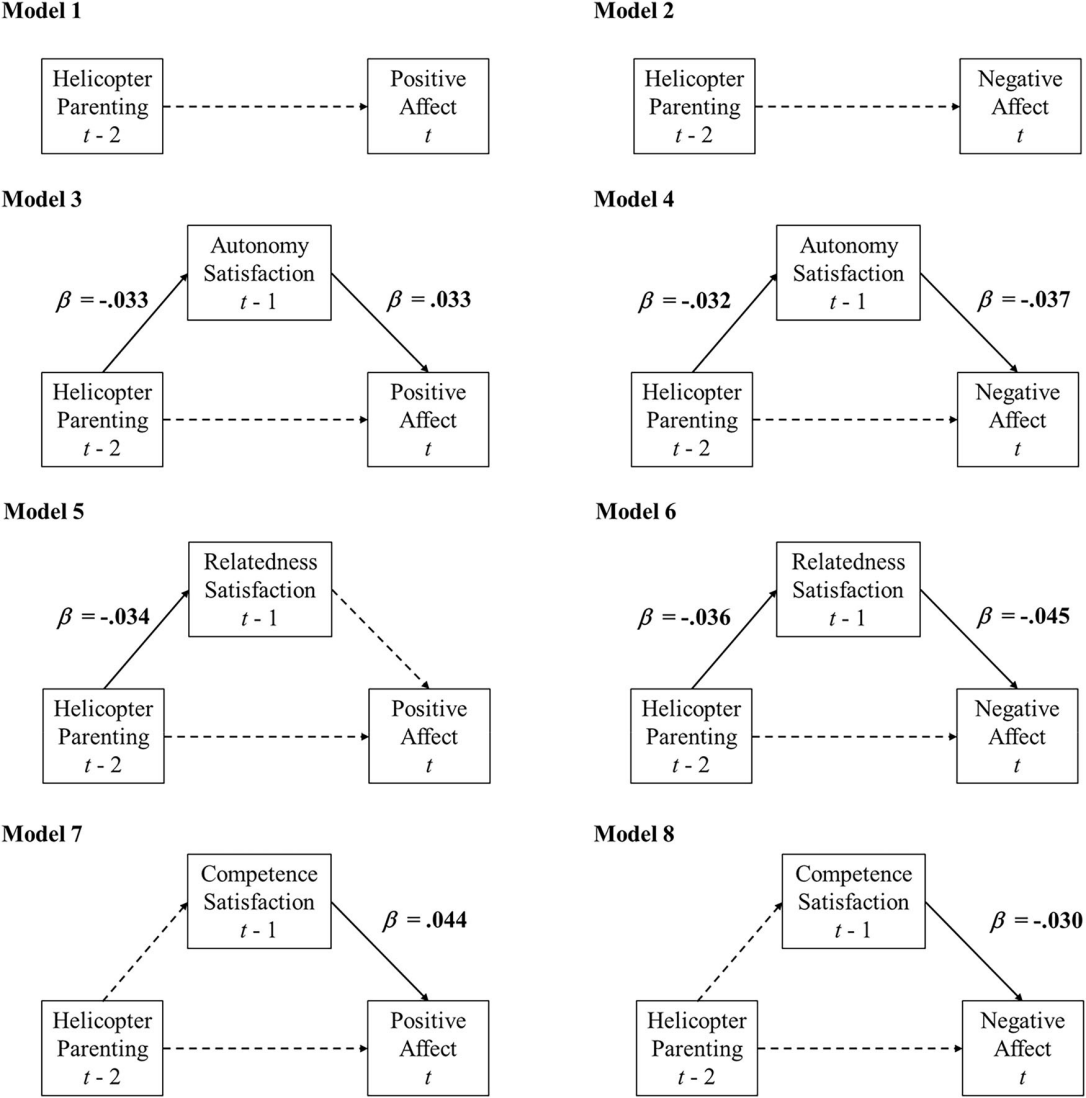
Within-Family Associations with Bi-weekly Time Intervals. *Note*: Dashed lines represent nonsignificant paths. Standardized coefficients are shown

As shown in Table 2 and Fig. 2, Model 1 and 2 examined the lagged effects of youth-reported helicopter parenting (at *t* -2) on youth’s positive and negative affect (at *t*). Results showed that the two lagged parenting effects were not significant, thus not supporting Hypothesis 1a and Hypothesis 1b. In other words, higher-than-usual levels of helicopter parenting did not directly predict changes in adolescents’ positive and negative affect four weeks later (i.e., two time units).

**Table 2:** Results of Models with Varying Timeframes (IN = 10,000, THIN = 2)

	Positive Affect (PA)			Negative Affect (NA)		
Average within-family	Est.	Est. St.	95%CI	Est.	Est. St.	95%CI
**Total Effects (Model 1-2)**						
Helicopter parenting (*t* - 2) → Affect (*t)*	−0.010	−0.009	[−0.045, 0.025]	0.009	0.008	[−0.020, 0.038]
**Autonomy Satisfaction (Model 3-4)**						
Helicopter parenting (*t* - 1) → Autonomy satisfaction (*t*)	−0.043*	−0.033	[−0.082, −0.005]	−0.042*	−0.032	[−0.081, −0.003]
Autonomy satisfaction (*t* - 1) → Affect (*t*)	0.026*	0.033	[0.003, 0.050]	−0.027*	−0.037	[−0.048, −0.007]
Helicopter parenting (*t* - 2) → Affect (*t)*	−0.009	−0.004	[−0.044, 0.025]	0.005	0.005	[−0.024, 0.036]
Helicopter parenting (*t* - 2) → Autonomy satisfaction (*t* - 1) →Affect (*t)*	−0.001	–	[−0.003, 0.000]	0.001*	–	[0.000, 0.003]
**Relatedness Satisfaction (Model 5-6)**						
Helicopter parenting (*t* - 1) → Relatedness satisfaction (*t*)	−0.045*	−0.034	[−0.084, −0.007]	−0.047*	−0.036	[−0.086, −0.009]
Relatedness satisfaction (*t* - 1) → Affect (*t*)	0.022	0.027	[−0.002, 0.046]	−0.033*	−0.045	[−0.055, −0.012]
Helicopter parenting (*t* - 2) → Affect (*t)*	−0.011	−0.009	[−0.045, 0.024]	0.007	0.007	[−0.023, 0.037]
Helicopter parenting (*t* - 2) → Relatedness satisfaction (*t* - 1) →Affect (*t)*	−0.001	–	[−0.003, 0.000]	0.001*	–	[0.000, 0.004]
**Competence Satisfaction (Model 7-8)**						
Helicopter parenting (*t* - 1) → Competence Satisfaction (*t*)	−0.034	−0.026	[−0.073, 0.003]	−0.036	−0.028	[−0.074, 0.002]
Competence Satisfaction (*t* - 1) → Affect (*t*)	0.035*	0.044	[0.010, 0.058]	−0.021*	−0.030	[−0.041, −0.002]
Helicopter parenting (*t* - 2) → Affect (*t)*	−0.004	−0.004	[−0.039, 0.030]	0.007	0.007	[−0.022, 0.037]
Helicopter parenting (*t* - 2) → Competence Satisfaction (*t* - 1) →Affect (*t)*	−0.001	–	[−0.003, 0.000]	0.001	–	[0.000, 0.002]

Parameters whose 95% credible interval does not contain zero are shown with an asterisk. Est = unstandardized estimate. Est. St. = standardized estimate (i.e., STDYX standardization). 95%CI = Bayesian Credible Intervals of unstandardized estimate. Not all parameter estimates are reported here and for full output see https://osf.io/qkza9/

### Indirect Effects through Psychological Need Satisfaction (*Hypothesis 2*)

#### Effects of helicopter parenting on psychological need satisfaction (Hypothesis 2a)

It was hypothesized that higher-than-usual levels of helicopter parenting (at *t* - 2) would predict decreases in adolescents’ psychological need satisfaction (at *t* -1). As predicted (see Table 2 and Fig. 2), Model 3 and Model 4 suggested that higher-than-usual levels of maternal helicopter parenting predicted decreases in adolescents’ autonomy (*β* = −0.033/−0.032) and relatedness satisfaction (*β* = −0.034/−0.036), but not competence satisfaction. In other words, adolescents reported lower autonomy and relatedness satisfaction, but unchanged competence satisfaction, after perceiving more maternal helicopter parenting behaviors two weeks earlier.

#### Effects of psychological need satisfaction on adolescent affect (Hypothesis 2c-d)

Models 3 to 8 examined the time-lagged effects of adolescents’ psychological need satisfaction (at *t* -1) on adolescent affect (at *t*) (see Table 2 and Fig. 2). Regarding positive affect, lower-than-usual levels of autonomy (*β* = 0.033) and competence satisfaction (*β* = 0.044) predicted decreases in adolescents’ positive affect. Relatedness satisfaction did not predict changes in positive affect. As predicted, lower-than-usual levels of all three psychological need satisfaction predicted increases in negative affect (−0.045 ≤ *β* ≤ −0.030), confirming Hypothesis 2 d. That is, adolescents reported less positive affect after having experienced reduced autonomy and competence need satisfaction two weeks earlier, and reported more negative affect after having experienced lower levels of each specific need satisfaction.

#### Indirect effects through psychological need satisfaction (Hypothesis 2g-h)

It was hypothesized that maternal helicopter parenting (at *t* -2) would predict adolescents’ later affect (at *t*) through each type of psychological need satisfaction (at *t* -1). That is, mediation pathways were hypothesized. As shown in Table 2, the indirect effects of helicopter parenting to negative affect through autonomy (unstandardized indirect effect = 0.001) and relatedness satisfaction (unstandardized indirect effect = 0.001) were significant, partly supporting Hypothesis 2 h. In other words, when adolescents perceived higher levels of maternal helicopter parenting than usual, their autonomy and relatedness satisfaction decreased two weeks later, which subsequently led to increases in their negative affect (Hypothesis 2 h) after another two weeks. These indirect processes were not observed for positive affect (thus not supporting Hypothesis 2 g) or for competence satisfaction.

### Exploratory Analyses

#### Mediation models with varying timeframes (preregistered)

To explore the timescale of the underlying dynamics in the associations between helicopter parenting, psychological need satisfaction, and affective well-being, two additional sets of timeframes were tested: a) youth-reported helicopter parenting (*t* - 1) was modeled to be correlated with adolescents’ psychological need satisfaction (*t* - 1), which, in turn, was modeled to predict adolescents’ affect after two weeks (*t*); b) youth-reported helicopter parenting was modeled to be correlated with adolescents’ psychological need satisfaction and adolescents’ affect at the same time point (*t*). See Table S5 for the sample sizes and tested paths for Models E1-E12.

**Fig. 3:**
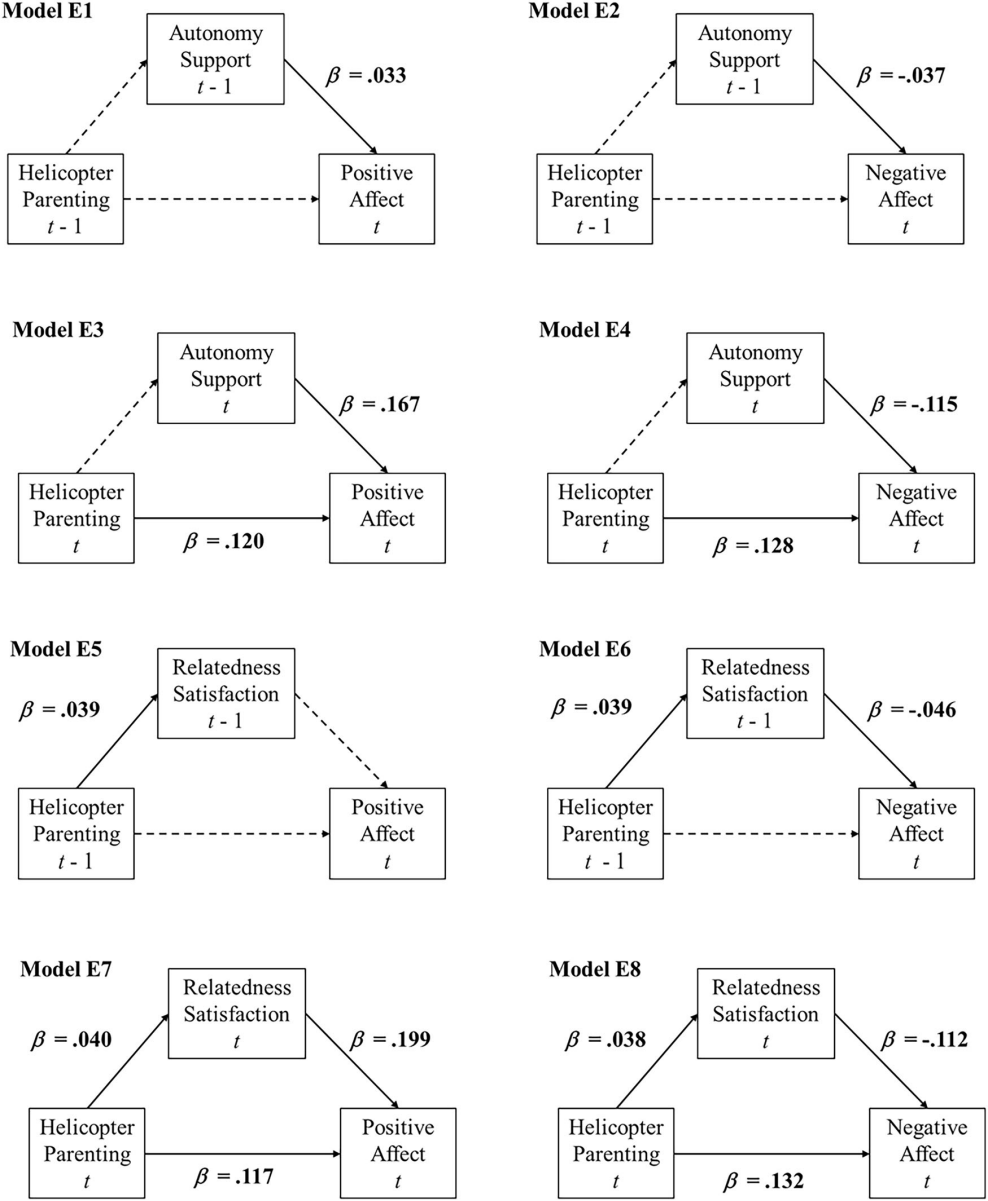
Within-Family Associations Examined with Varying Timeframes. *Note*: This figure represents models: (a) helicopter parenting (*t* - 1) → autonomy/relatedness satisfaction (*t* - 1) → affective well-being (*t*), and (b) helicopter parenting (*t*) → autonomy/relatedness satisfaction (*t*) → affective well-being (*t*). Models regarding competence satisfaction are present in Figure S3. Dashed lines represent nonsignificant paths. Standardized coefficients are shown

Regarding autonomy satisfaction (see Models E1-E4 in Table 3, Fig. 3, and Figure S1), Models E1 and E2 showed no direct effects of helicopter parenting (at *t* -1) on adolescents’ positive and negative affect (at *t*) two weeks later. Helicopter parenting were not concurrently associated with autonomy satisfaction. Though adolescents’ lower-than-usual levels of autonomy satisfaction predicted decreased positive affect (*β* = 0.033) and increased negative affect (*β* = −0.037), the indirect effects were not established. For concurrent modeling, Model E3 and E4 suggested that helicopter parenting was positively associated with positive affect (*β* = 0.120) and negative affect (*β* = 0.128). That is, adolescents reported more positive and negative affect than usual when they perceived higher-than-usual levels of helicopter parenting behaviors. Though lower-than-usual levels of adolescents’ autonomy satisfaction were concurrently related to decreased positive affect (*β* = 0.167) and increased negative affect (*β* = −0.115), helicopter parenting was not concurrently linked to adolescents’ autonomy satisfaction, thus the indirect effects assessing the concurrent phenomena were not significant.

**Table 3:** Results of Exploratory Analyses with Different Temporal Order (IN = 10,000, THIN = 2)

	Autonomy Satisfaction (Model E1-E4)					
	Positive Affect (PA)			Negative Affect (NA)		
Average within-family	Est.	Est. St.	95%CI	Est.	Est. St.	95%CI
Explanatory Temporal Order 1 (Model E1-E2)						
Helicopter parenting (*t*) → Autonomy satisfaction (*t*)	0.001	0.001	[−0.036, 0.039]	0.000	0.000	[−0.038, 0.037]
Autonomy satisfaction (*t* - 1) → Affect (*t*)	0.026*	0.033	[0.003, 0.050]	−0.027*	−0.037	[−0.047, −0.006]
Helicopter parenting (*t* - 1) → Affect (*t)*	0.005	0.004	[−0.029, 0.039]	0.008	0.008	[−0.020, 0.037]
Helicopter parenting (*t* - 1) → Autonomy satisfaction (*t* - 1) → Affect (*t)*	0.000	–	[−0.001, 0.001]	0.000	–	[−0.001, 0.001]
Explanatory Temporal Order 2 (Model E3-4)						
Helicopter parenting (*t*) → Autonomy satisfaction (*t*)	0.002	0.001	[−0.035, 0.039]	−0.001	−0.001	[−0.038, 0.037]
Autonomy satisfaction (*t*) → Affect (*t*)	0.134*	0.167	[0.113, 0.156]	−0.087*	−0.115	[−0.108, −0.065]
Helicopter parenting (*t*) → Affect (*t)*	0.139*	0.120	[0.107, 0.171]	0.140*	0.128	[0.108, 0.171]
Helicopter parenting (*t*) → Autonomy satisfaction (*t*) → Affect (*t)*	0.000	-	[−0.005, 0.005]	0.000	-	[−0.003, 0.003]

	Relatedness Satisfaction (Model E5-E8)					
	Positive Affect (PA)			Negative Affect (NA)		
Average within-family	Est.	Est. St.	95%CI	Est.	Est. St.	95%CI
Explanatory Temporal Order 1 (Model E5-E6)						
Helicopter parenting (*t*) → Relatedness satisfaction (*t*)	0.051*	0.039	[0.013, 0.088]	0.051*	0.039	[0.013, 0.088]
Relatedness satisfaction (*t* - 1) → Affect (*t*)	0.021	0.027	[−0.003, 0.046]	−0.034*	−0.046	[−0.056, −0.012]
Helicopter parenting (*t* - 1) → Affect (*t)*	0.005	0.004	[−0.030, 0.038]	0.014	0.013	[−0.013, 0.042]
Helicopter parenting (*t* - 1) → Relatedness satisfaction (*t* - 1) → Affect (*t)*	0.001	–	[0.000, 0.003]	−0.002*	–	[−0.004, 0.000]
Explanatory Temporal Order 2 (Model E7-E8)						
Helicopter parenting (*t*) → Relatedness satisfaction (*t*)	0.052*	0.040	[0.015, 0.090]	0.050*	0.038	[0.012, 0.088]
Relatedness satisfaction (*t*) → Affect (*t*)	0.161*	0.199	[0.139, 0.183]	−0.084*	−0.112	[−0.105, −0.063]
Helicopter parenting (*t*) → Affect (*t)*	0.137*	0.117	[0.104, 0.169]	0.142*	0.132	[0.112, 0.173]
Helicopter parenting (*t*) → Relatedness satisfaction (*t*) → Affect (*t)*	0.008*	–	[0.002, 0.015]	−0.004*	–	[−0.008, −0.001]

	Competence Satisfaction (Model E9-E12)					
	Positive Affect (PA)			Negative Affect (NA)		
Average within-family	Est.	Est. St.	95%CI	Est.	Est. St.	95%CI
Explanatory Temporal Order 1 (Model E9-E10)						
Helicopter parenting (*t*) → Competence Satisfaction (*t*)	0.027	0.020	[−0.008, 0.063]	0.026	0.020	[−0.011, 0.063]
Competence Satisfaction (*t* - 1) → Affect (*t*)	0.035*	0.046	[0.011, 0.058]	−0.022*	−0.031	[−0.042, −0.002]
Helicopter parenting (*t* - 1) → Affect (*t)*	0.004	0.004	[−0.031, 0.039]	0.012	0.012	[−0.016, 0.040]
Helicopter parenting (*t* - 1) → Competence Satisfaction (*t* - 1) → Affect (*t)*	0.001	–	[0.000, 0.003]	0.000	–	[−0.002, 0.000]
Explanatory Temporal Order 2 (Model E11-E12)						
Helicopter parenting (*t*) → Competence Satisfaction (*t*)	0.028	0.021	[−0.009, 0.065]	0.025	0.019	[−0.012, 0.062]
Competence Satisfaction (*t*) → Affect (*t*)	0.190*	0.242	[0.169, 0.211]	−0.115*	−0.159	[−0.136, −0.095]
Helicopter parenting (*t*) → Affect (*t)*	0.133*	0.114	[0.101, 0.166]	0.143*	0.133	[0.113, 0.174]
Helicopter parenting (*t*) → Competence Satisfaction (*t*) → Affect (*t)*	0.005	–	[−0.002, 0.012]	−0.003	–	[−0.007, 0.001]

Parameters whose 95% credible interval does not contain zero are shown with an asterisk. Est = unstandardized estimate. Est. St. = standardized estimate (i.e., STDYX standardization). 95%CI = Bayesian Credible Intervals of unstandardized estimate. Not all parameter estimates are reported here; for full output, see (https://osf.io/qkza9/)

Examination of relatedness satisfaction (see Models E5-E8 in Table 3, Fig. 3, and Figure S2) suggested that the time intervals of the model mattered. Specifically, in models without a time lag (assessing concurrent phenomena, Models E5-E8 in Figure S2), higher-than-usual levels of helicopter parenting had links with *increases* in relatedness satisfaction, suggesting that they positively waxed and waned together. However, when the time lag became larger, higher-than-usual levels of helicopter parenting predicted *decreased* relatedness satisfaction after two weeks (as shown in Model 5–6 in Table 2 and Fig. 2). Regarding the indirect effect of helicopter parenting through relatedness satisfaction on positive affect, it was only established when assessing concurrent processes. For negative affect, the indirect effects were established at all timeframes, but the valence of indirect effects with a two-week time lag between helicopter parenting and relatedness satisfaction (Model 5–6) were opposite to the valence of the concurrent links (Model E5-E8).

No significant associations were observed between helicopter parenting and competence satisfaction, either concurrently or with a two-week time lag (see Models E9-E12 in Table 3 and Figure S3). Hence, the mediating role of need satisfaction in the links between helicopter parenting and youth’s poorer well-being appeared to mainly pertain to autonomy needs, and to a lesser extent relatedness needs (for which the dynamics might be timescale-specific).

#### Helicopter parenting dimensions (Exploratory)

Exploratory analyses linking different helicopter parenting dimensions to the satisfaction of each specific need (see Models E13-E24 in Table S6 for sample sizes) were also conducted. Results showed that both Advice/Affect Management and Anticipatory Problem Solving negatively predicted youth’s autonomy satisfaction, only Emphasis on Academic Achievement negatively predicted relatedness satisfaction, and no helicopter parenting dimension predicted competence satisfaction (see Table S7 in the supplemental materials for more details).

#### Mother-reported helicopter parenting (Exploratory)

Exploratory analyses using mother-reported helicopter parenting (see Models E25-E32 in Table S8 for sample sizes) indicated that increased mother-reported helicopter parenting predicted higher-than-usual levels of youth negative affect over time. The indirect effects through various dimensions of youth’s psychological need satisfaction were not significant (see Table S9 in the supplemental materials for more details).

## Discussion

Although youth’s psychological need satisfaction has been documented to explain the links between helicopter parenting and poorer youth outcomes, the time-lagged nature of these processes has not yet been examined within families. Analyzing 5,357 reports from 350 late adolescents, this study applied intensive longitudinal mediation models to test these processes, providing support for the applicability of SDT in within-family processes. Though the total effects of helicopter parenting on youth affect after four weeks were not supported, the findings revealed indirect effects of helicopter parenting on youth affect through autonomy and relatedness satisfaction at bi-weekly intervals. Moreover, the associations between helicopter parenting, psychological need satisfaction, and affective well-being varied depending on the interval in question, indicating the dynamics changed both in valence and statistical significance over time.

### Psychological Need Satisfaction: Linking Parenting and Youth Well-Being

SDT (Ryan & Deci, 2017; Soenens et al., 2017) suggests that helicopter parenting reduces satisfaction of adolescents’ psychological needs and is thus detrimental to their well-being. These processes should occur within families and unfold over time. The present study is the first to assess these proposed underlying within-family processes at meso-timescales (i.e., two weeks). Although no significant total effects of helicopter parenting on youth affective well-being (Hypothesis 1) were observed at either two-week or four-week time lags, the hypothesized indirect effects were indeed present. Partly supporting Hypothesis 2, results showed detrimental effects of helicopter parenting on adolescents’ affective well-being via psychological need satisfaction, particularly autonomy and relatedness satisfaction. These results highlight the applicability of SDT in parenting contexts (Soenens et al., 2017; Soenens et al., 2015), suggesting that satisfaction of specific psychological needs can serve as a link between parenting practices and youth well-being. Though the observed effects were small, these findings are still meaningful when considering development as a dynamic system. Specifically, frequent positive affect has been associated with success in various life domains (Lyubomirsky et al., 2005), and prolonged and repeated experiences of negative moods can cumulatively contribute to the development of emotional difficulties (Stefanovic et al., 2021). Thus, small detrimental effects at bi-weekly intervals might point to a more stable state dysfunction that necessitates intervention.

### Unique Effects of Specific Psychological Needs

The present research further highlights the different roles of each psychological need proposed by SDT (Schiffrin et al., 2019; Soenens et al., 2017) in explaining the links between helicopter parenting and affective well-being. Within families, helicopter parenting held diverse associations with different basic needs, with notable time-lagged negative impacts on youth’s satisfaction of autonomy and relatedness, but not competence. These detrimental effects on autonomy and relatedness satisfaction likely reflect the combination of overly demanding and overly responsive characteristics of helicopter parenting (Hong & Cui, 2023; Padilla-Walker & Nelson, 2012). Late adolescents’ and emerging adults’ need for autonomy can be threatened when they desire the ability to make independent choices but still experience excessive parental involvement. Helicopter parents who invest time, money, and energy in their children’s lives possibly hold the belief that they “have a say” in their children’s decision-making processes (Lowe et al., 2015) and expect obedience and compliance from their children. Adolescents’ need for relatedness can be additionally threatened when, despite parents’ frequent involvement, they view this support as contingent on their loyalty and obedience, ultimately leading to a low-quality parent-youth bond (Soenens et al., 2017).

Prior studies have suggested that helicopter parenting might threaten youth’s need for competence (e.g., Hong & Cui, 2023; Schiffrin et al., 2019). No within-family associations existed between helicopter parenting and competence satisfaction, however, either concurrently or with a two-week lag. These results might indicate a limited impact of helicopter parenting on youth’s sense of competence in late adolescence and emerging adulthood; alternatively, the dynamic interplay between helicopter parenting and competence satisfaction might occur at other timescales, either shorter or longer. Future studies could investigate whether the associations between helicopter parenting and competence satisfaction are more pronounced during early adolescence, or at different timescales.

Exploration of each helicopter parenting dimension (Segrin et al., 2013; Zong & Hawk, 2022) suggested that higher-than-usual levels of Advice/Affect Management and Anticipatory Problem Solving preceded within-person decreases in autonomy satisfaction. Additionally, higher levels of Emphasis on Academic Performance were followed by decreased relatedness satisfaction. Bi-weekly changes in each dimension of helicopter parenting did not predict later changes in competence satisfaction. These findings add nuance to the links between helicopter parenting dimensions and specific psychological needs, supporting the multidimensionality of helicopter parenting. Future studies could benefit from examining these processes in greater detail. From a practical standpoint, parents should intentionally reduce unnecessary involvement, especially those that could impede their college-age children’s need satisfaction, as the present findings indicate that need satisfaction exhibits stable and robust protective effects on youth affective well-being both concurrently and after weeks.

Exploratory analyses using mother-reported helicopter parenting revealed a different pattern. Specifically, mother-reported helicopter parenting had direct detrimental effects on youth affect that were not mediated by psychological need satisfaction. These findings are consistent with previous studies using dyadic youth-parent reports suggesting that youths’ own perceptions of parenting practices are more closely aligned with their psychological need satisfaction (see also in Van Petegem et al., 2020). Despite the inconsistency of the indirect effects, these findings still point to the long-term detrimental effects of helicopter parenting within Chinese families, regardless of the informant.

### Time Intervals Matter

Established theories and empirical studies (Boele et al., 2023; Lougheed, 2020) assume that developmental processes occur and demonstrate varying effects at different time intervals. The current study supports this claim by identifying differences in valence and statistical significance between concurrent and time-lagged associations. Regarding affective well-being, significant concurrent (but not time-lagged) associations existed between youth-reported helicopter parenting and adolescent affect, suggesting time-dependent processes. Helicopter parenting also exhibited concurrent positive associations with both positive and negative affect. These results align with a prior study, conducted with the same sample, that identified positive within-family associations between helicopter parenting and both beneficial (i.e., support) and detrimental (i.e., conflict) aspects of youth-mother relationship quality (Wang & Hawk, 2023). When experiencing higher-than-usual levels of helicopter parenting, youth might experience a combination of relief stemming from parents solving their everyday challenges, and distress stemming from perceptions that parents are intruding into their personal matters. The effects of helicopter parenting could then diminish in the weeks that follow, as youth adapt to their parents’ excessive involvement and adjust their affect accordingly.

In contrast, the impact of helicopter parenting on youth’s deeper psychological needs might become more pronounced as time progresses. Results showed that youth-reported helicopter parenting was not concurrently associated with autonomy satisfaction, but it was followed by a decrease in autonomy satisfaction at the next measurement. These results could indicate that parenting effects on autonomy satisfaction accumulate over time and manifest at the meso-timescale (e.g., bi-weekly). Results also showed a shift in valence, where higher-than-usual levels of helicopter parenting were initially associated with concurrent increases in relatedness satisfaction, but also subsequent decreases in relatedness satisfaction after two weeks. These results might suggest that a higher *quantity* of parental involvement strengthens the youth-parent bond in the shorter term, because parents’ timely and intensive interventions to youth’s difficulties foster strong feelings of support. As time goes on, however, the *quality* of parental involvement could become more salient, and adolescents start questioning whether their parents’ excessive assistance truly meets their needs or, conversely, is primarily driven by their parents’ own needs or expectations. These results convey an important message for parents and practitioners, as parenting approaches that initially demonstrate harmless or even immediately beneficial characteristics can still have certain detrimental effects over longer periods (Keijsers et al., 2022). This pattern might be particularly salient during the transition to college, a critical turning point when youth in Hong Kong typically expect and achieve greater independence (e.g., living apart from parents) following an extended period of compulsory education and competitive college entrance exams. Future studies with reports from multiple family members could yield valuable insights into how parents and adolescents best cope with this transition and the adolescents’ developing autonomy.

In sum, these findings highlight the complexity of parenting processes, as parenting effects can be both time-dependent and variable-specific. These results align with previous studies highlighting the qualitative and quantitative differences in the underlying dynamics driving long-term development at multiple timescales (Boele et al., 2023; Bülow et al., 2023), and underscore the conceptual and methodological necessity of differentiating and comparing parenting effects within various timescales (e.g., Keijsers & Van Roekel, 2018). Future studies on helicopter parenting would benefit from investigating within-family processes across several different timescales, ranging from daily to yearly and beyond, to explore whether the bi-weekly processes observed here are cumulative effects of relatively micro-timescale (e.g., daily) processes, and whether the accumulation of bi-weekly effects ultimately leads to more severe long-term difficulties (Lougheed, 2020).

### Limitations and Future Directions

As the first meso-longitudinal study to assess the bi-weekly effects of helicopter parenting on need satisfaction and affective well-being, these findings underscore the significance of aligning research questions and methods to effectively capture and understand parenting processes (Boele et al., 2020; Keijsers, 2016). Even though this study addresses methodological and theoretical limitations of earlier work, it also comes with its own caveats. First, stationary mediation models were used due to the limited number of time points (i.e., 16 time points). This is a preliminary approach to examining intensive longitudinal mediation where the indirect effect is modeled as being constant over both time and people (Huang & Yuan, 2017; McNeish & MacKinnon, 2022). However, recent research has highlighted that parenting effects could vary substantially between families (e.g., Boele et al., 2024; Bülow, Neubauer, et al., 2022), indicating that the observed average within-family results might not necessarily apply to all families. For example, some studies suggest that Chinese mothers exhibit greater protectiveness and management toward girls (e.g., Wu et al., 2023), which could result in more pronounced negative outcomes. The possibility of effect heterogeneity across families and the potential moderating effects of individual factors calls for future research to expand upon these findings using person-specific models, dynamic models, or even cross-classified models where the indirect effects can vary across time and/or individuals (McNeish & MacKinnon, 2022).

Second, the meso-longitudinal approach is a relatively new research design (Boele et al., 2020) and many instruments to assess weekly fluctuations are still in development. The present study did not examine the specific mediating role of psychological need frustration, compared to need satisfaction, due to potential reliability and representativeness issues arising from the use of the single-item measure (Allen et al., 2022). Future research should continue to develop and employ more reliable measurement tools to investigate whether psychological need satisfaction and frustration have differential effects on youths’ positive and negative affect.

Third, the present study is based on a relatively homogenous adolescent sample, with all participants being first-year college students at the same university. Even though participants were from diverse academic tracks, caution should be exercised when interpreting the results and future research should aim to improve generalizability by including a more diverse sample. Finally, the data were collected during the COVID-19 pandemic, which potentially influenced both parenting behaviors, youth well-being, and family relationships (see meta-analyses and systematic reviews: Campione-Barr et al., 2024; Wolf & Schmitz, 2024). Specifically, social distancing regulations in Hong Kong required youth to reside at home rather than on campus, potentially maintaining or increasing interactions with mothers in the pivotal period of transitioning to college. Mothers might also have responded to this public health crisis by becoming more involved compared to pre-pandemic conditions, to ensure their children’s safety; youth, already in a more vulnerable state of reduced autonomy, might have experienced altered effects of parenting. Replications are still needed to establish the generalizability of these findings in post-COVID contexts and to clarify the potential impacts of COVID-19 on parenting processes.

## Conclusion

Earlier work based on Self-Determination Theory had suggested that decreases in adolescents’ psychological need satisfaction could explain why helicopter parenting is linked to poorer adolescent outcomes across cultures. By applying intensive mediation modeling, this preregistered study investigated the underlying dynamics among Chinese late adolescents by assessing the temporal sequence of these theorized processes within families on a bi-weekly basis. Results showed that maternal helicopter parenting, in the short term, can increase both positive and negative affect. When examined over the course of several weeks, however, higher-than-usual levels of helicopter parenting predicted decreases in adolescents’ autonomy and relatedness (but not competence) satisfaction after two weeks, subsequently leading to declines in their affective well-being after another two weeks. As theorized, helicopter parenting appears to reduce opportunities for youth to experience psychological need satisfaction, which ultimately undermines their well-being. These processes are need-specific and time-independent, highlighting the complexity of parenting dynamics.

## Supplementary information

Supplemental Materials
