# Supplementary material for: Helicopter Parenting and Youth Affective Well-Being: Need Satisfaction as a Within-Family Mediator
Source: J Youth Adolesc. 2025 Mar 18;54(8):1917–33. doi: 10.1007/s10964-025-02164-1 (PMC12331812; doi:10.1007/s10964-025-02164-1)
Supplement: Supplementary file 1 — Supplemental Materials [file 10964_2025_2164_MOESM1_ESM.docx]

Supplemental Materials for

**Helicopter Parenting and Youth Affective Well-being: Need Satisfaction as a Within-Family Mediator**

Table of Contents

[1. Measures 2](#_Toc184037537)

[1.1. Helicopter Parenting Behavior (Bi-weekly, adolescent-report as example) 2](#_Toc184037538)

[1.2. Positive and Negative Affect (Bi-weekly) 4](#_Toc184037539)

[1.3. Psychological need satisfaction and frustration (Bi-weekly) 5](#_Toc184037540)

[2. Multilevel confirmatory factor analysis 7](#_Toc184037541)

[2.1. Helicopter parenting 7](#_Toc184037542)

[2.2. Positive and negative affect 8](#_Toc184037543)

[2.3. Psychological need satisfaction 9](#_Toc184037544)

[3. Sample size and examined paths for main models. 10](#_Toc184037545)

[4. Exploratory analyses 11](#_Toc184037546)

[4.1. Exploratory analyses with different temporal orders 11](#_Toc184037547)

[4.1.1. Participants without within-person variance (ID) 11](#_Toc184037548)

[4.1.2. Figures For Explanatory Analyses with Different Needs 12](#_Toc184037549)

[4.2. Exploratory analyses linking different helicopter parenting dimensions with different psychological needs 15](#_Toc184037550)

[4.2.1. Participants without within-person variance (ID) 15](#_Toc184037551)

[4.2.2. Results For Explanatory Analyses with Different Helicopter Parenting Dimensions and Different Psychological Needs 17](#_Toc184037552)

[4.3. Exploratory analyses using mother-reported helicopter parenting 18](#_Toc184037553)

[4.3.1. Participants without within-person variance (ID) 18](#_Toc184037554)

[4.3.2. Results For Explanatory Analyses using Mother-reported Helicopter Parenting 19](#_Toc184037555)

1. Measures
   1. Helicopter Parenting Behavior (Bi-weekly, adolescent-report as example)

*In the past week, how often has you MOTHER…*

hpb1: Given you advice about how to do things.

建議你如何處理事情。

hpb2: Made suggestions to help you get things accomplished.

給你建議來幫助你完成事情。

hpb3: Said or done something to cheer you up.

說或做些事情來使你振作起來。

hpb4: Made special efforts to make you happy.

竭盡全力令你開心。

hpb5: Tried to solve a problem for you before you even experienced it.

在你還未經歷某些問題前就試圖為你解決。

hpb6: Tired to help you steer clear a difficulty that you might have otherwise encounter.

試圖協助你避開任何你可能會遇到的煩惱。

hpb7: Asked for “behind the scenes” information about your social life.

問及你社交生活的大小細節。

hpb8: Asked for updates about your schedule, whereabouts, and day-to-day activities.

問及你每日的行程、行蹤及日常生活的情況。

hpb9: Used social media to follow your day-to-day activities.

利用社交媒體追蹤你的情況。

hpb10: Made a specific effort to raise your academic results.

竭盡全力來提高你的學業成績。

hpb11: Paid strong attention to your school assignments and/or exams.

非常重視你學校的功課及／或考試。

1 = Not at all

2 = Rarely

3 = Occasionally

4 = Often

5 = Very often

Subscales: Advice/Affect Management (hp1-hp4); Anticipatory Problem Solving (hp5-hp6); Information Seeking (hp7-hp9); Emphasis on Academic Performance (hp10-hp11)

- 1. Positive and Negative Affect (Bi-weekly)

*How often in the past week have you felt…*

sa4: Happy. 開心

sa5: Relaxed. 輕鬆

sa7: Sad. 傷心

sa8: Afraid. 恐懼

sa9: Hostile. 對人有敵意

sa10: Anxious. 焦慮不安

sa11: Energetic. 充滿能量

sa14: Short-tempered. 脾氣暴躁

sa15: Cheerful. 快樂

sa21: Motivated. 有動力

sa22: Depressed. 抑鬱

1 = Not at all

2 = Rarely

3 = Occasionally/ Sometimes

4 = Often

5 = Very often

Subscales: Positive Affect (sa4, sa5, sa11, sa15, and sa21); Negative Affect (sa7, sa8, sa9, sa10, sa14, and sa22).

*Note*: The full scale and validation of this shorten scale can be found in https://osf.io/qkza9/?view_only=64f3f9f8445c4035a57699bc8827d416

- 1. Psychological need satisfaction and frustration (Bi-weekly)

*Please indicate your current level of agreement with the following statements:*

sdt1: I feel a sense of choice and freedom in the things I undertake.

對於我從事的事情，我感到有個人的選擇及自由。

sdt2: I feel pressured to do too many things.

我感到有壓力要去做很多事情。

sdt3: I feel that my decisions reflect what I really want.

我感到我的決定均能反映我真正想要的。

sdt4: I feel that the people I care about also care about me.

我感到我所關心的人也都關心我。

sdt5: I feel the relationships I have are just superficial.

我感到我的關係都是表面的。

sdt6: I feel close and connected with other people who are important to me.

我感到與對我重要的人之間有親密的聯繫。

sdt7: I feel confident that I can do things well.

我有信心可以把事情做好。

sdt8: I feel capable at what I do.

我感到自己有能力處理要做的事情。

sdt9: I feel insecure about my abilities.

我對於自己的能力不太肯定。

1 = Very untrue of me

2 = Untrue of me

3 = Mildly untrue of me

4 = Mildly true of me

5 = Ture of me

6 = Very true of me

Subscales: Need Satisfaction (sdt1, sdt3, sdt4, sdt6, sdt7, and sdt8); Need Frustration (sdt2, sdt5, and sdt9).

1. Multilevel confirmatory factor analysis
   1. Helicopter parenting

| Table S1 |  | |
| --- | --- | --- |
| *Standardized Factor Loading* |  | |
| Item | Within | Between |
| **Affect/Advice Management** |  |  |
| hpb1: Given you advice about how to do things. | .58 | .95 |
| hpb2: Made suggestions to help you get things accomplished. | .64 | 1.01 |
| hpb3: Said or done something to cheer you up. | .57 | .73 |
| hpb4: Made special efforts to make you happy. | .46 | .54 |
| **Anticipatory Problem Solving** |  |  |
| hpb5: Tried to solve a problem for you before you even experienced it. | .83 | 1.00 |
| hpb6: Tired to help you steer clear a difficulty that you might have otherwise encounter. | .80 | .99 |
| **Information Seeking** |  |  |
| hpb7: Asked for “behind the scenes” information about your social life. | .79 | 1.03 |
| hpb8: Asked for updates about your schedule, whereabouts, and day-to-day activities. | .72 | .87 |
| hpb9: Used social media to follow your day-to-day activities. | .27 | .49 |
| **Emphasis on Academic Performance** |  |  |
| hpb10: Made a specific effort to raise your academic results. | .77 | 1.01 |
| hpb11: Paid strong attention to your school assignments and/or exams. | .62 | .87 |

- 1. Positive and negative affect

| Table S2 |  | |
| --- | --- | --- |
| *Standardized Factor Loading* |  | |
| Item | Within | Between |
| **Positive Affect** |  |  |
| Happy | .69 | .96 |
| Relaxed | .61 | .82 |
| Energetic | .56 | .87 |
| Cheerful | .71 | .99 |
| Motivated | .46 | .80 |
| **Negative Affect** |  |  |
| Sad | .60 | .92 |
| Afraid | .64 | .89 |
| Hostile | .46 | .82 |
| Anxious | .63 | .80 |
| Short-tempered | .42 | .82 |
| Depressed | .57 | .92 |

- 1. Psychological need satisfaction

| Table S3 |  |  |
| --- | --- | --- |
| *Standardized Factor Loading for Need Satisfaction – 3 Factor* | | |
| item | Within | Between |
| **Autonomy satisfaction** |  |  |
| I feel a sense of choice and freedom in the things I undertake | .57 | .93 |
| I feel that my decisions reflect what I really want | .62 | .86 |
| **Relatedness Satisfaction** |  |  |
| I feel that the people I care about also care about me | .64 | .99 |
| I feel close and connected with other people who are important to me | .63 | .94 |
| **Competence Satisfaction** |  |  |
| I feel confident that I can do things well | .79 | .99 |
| I feel capable at what I do | .78 | 1.00 |

*Note*: A = Autonomy, C = Competence, R = relatedness.

3. Sample size and examined paths for main models.

Helicopter parenting (HP, 2): 223, 265

Positive affect (PA, 1): 75

Negative affect (NA, 1): 219

Autonomy Satisfaction (AS, 4): 75, 79, 176, 222

Relatedness Satisfaction (RS, 7): 41, 54, 75, 79, 93, 176, 244

Competence Satisfaction (CS, 11): 10, 41, 57, 76, 79, 130, 175, 176, 189, 222, 226

| Table S4 | | |  |
| --- | --- | --- | --- |
| *Sample Size for Analyses with Different Psychological Needs* | | | |
| Model | Path(s) | Excluded participants | *n* |
| Total Effect |  |  |  |
| Model 1 | HP (*t* - 2) 🡪 PA (*t*) | 3 | 347 |
| Model 2 | HP (*t* - 2) 🡪 NA (*t*) | 3 | 347 |
| Need for Autonomy | | |  |
| Model 3 | HP (*t* - 2) 🡪 AS (*t* - 1) 🡪 PA (*t*) | 6 | 344 |
| Model 4 | HP (*t* - 2) 🡪 AS (*t* - 1) 🡪 NA (*t*) | 7 | 343 |
| Need for Relatedness | |  |  |
| Model 5 | HP (*t* - 2) 🡪 RS (*t* - 1) 🡪 PA (*t*) | 9 | 341 |
| Model 6 | HP (*t* - 2) 🡪 RS (*t* - 1) 🡪 NA (*t*) | 10 | 340 |
| Need for competence | |  |  |
| Model 7 | HP (*t* - 2) 🡪 CS (*t* - 1) 🡪 PA (*t*) | 14 | 336 |
| Model 8 | HP (*t* - 2) 🡪 CS (*t* - 1) 🡪 NA (*t*) | 14 | 336 |

*Note*: HP = helicopter parenting, PA = positive affect, NA = negative affect, PNS = psychological need satisfaction, AS = Autonomy Satisfaction, RS = Relatedness Satisfaction, CS = Competence Satisfaction.

1. Exploratory analyses
   1. Exploratory analyses with different temporal orders
      1. Participants without within-person variance (ID)

Helicopter parenting (HP, 2): 223, 265

Positive affect (PA, 1): 75

Negative affect (NA, 1): 219

Autonomy Satisfaction (AS, 4): 75, 79, 176, 222

Relatedness Satisfaction (RS, 7): 41, 54, 75, 79, 93, 176, 244

Competence Satisfaction (CS, 11): 10, 41, 57, 76, 79, 130, 175, 176, 189, 222, 226

| Table S5 | | |  |
| --- | --- | --- | --- |
| *Sample Size for Exploratory Analyses with Different Psychological Needs* | | | |
| Model | Path(s) | Excluded participants | *n* |
| Need for Autonomy | | |  |
| Model E1 | HP (*t* - 1) 🡪 AS (*t* - 1) 🡪 PA (*t*) | 6 | 344 |
| Model E2 | HP (*t* - 1) 🡪 AS (*t* - 1) 🡪 NA (*t*) | 7 | 343 |
| Model E3 | HP (*t*) 🡪AS (*t*) 🡪 PA (*t*) | 6 | 344 |
| Model E4 | HP (*t*) 🡪 AS (*t*) 🡪 NA (*t*) | 7 | 343 |
| Need for Relatedness | |  |  |
| Model E5 | HP (*t* - 1) 🡪 RS (*t* - 1) 🡪 PA (*t*) | 9 | 341 |
| Model E6 | HP (*t* - 1) 🡪 RS (*t* - 1) 🡪 NA (*t*) | 10 | 340 |
| Model E7 | HP (*t*) 🡪RS (*t*) 🡪 PA (*t*) | 9 | 341 |
| Model E8 | HP (*t*) 🡪 RS (*t*) 🡪 NA (*t*) | 10 | 340 |
| Need for competence | |  |  |
| Model E9 | HP (*t* - 1) 🡪 CS (*t* - 1) 🡪 PA (*t*) | 14 | 336 |
| Model E10 | HP (*t* - 1) 🡪 CS (*t* - 1) 🡪 NA (*t*) | 14 | 336 |
| Model E11 | HP (*t*) 🡪 CS (*t*) 🡪 PA (*t*) | 14 | 336 |
| Model E12 | HP (*t*) 🡪 CS (*t*) 🡪 NA (*t*) | 14 | 336 |

*Note*: HP = helicopter parenting, PA = positive affect, NA = negative affect, PNS = psychological need satisfaction, AS = Autonomy Satisfaction, RS = Relatedness Satisfaction, CS = Competence Satisfaction.

- - 1. Figures For Explanatory Analyses with Different Needs
       1. Need for autonomy.

Figure S1

*Within-Family Models Testing Autonomy Satisfaction (AS) as the Mediator at Different Temporal Orders*


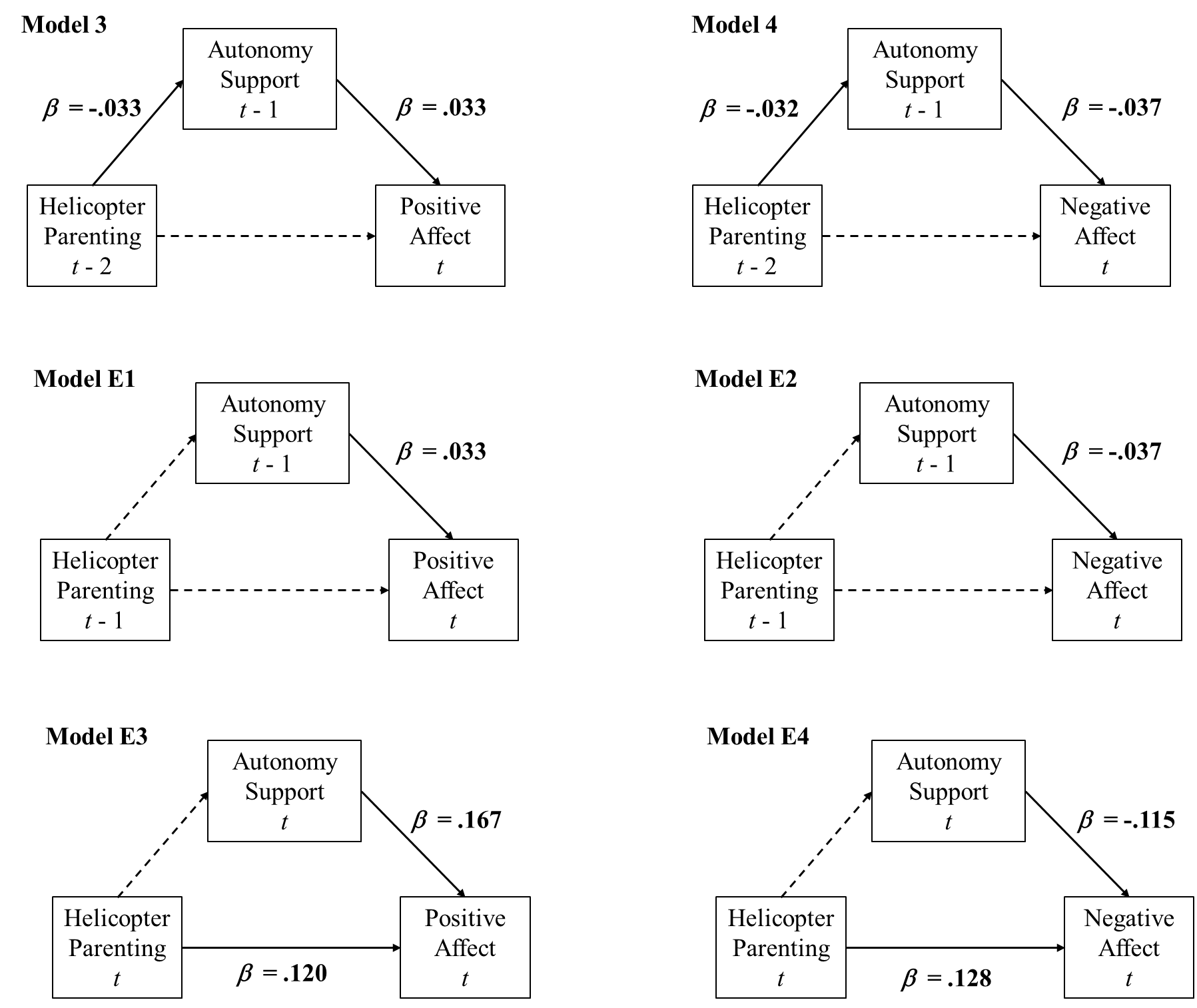


*Note.* This figure represents the mediation models: (A) helicopter parenting (*t* - 2) 🡪 autonomy satisfaction (*t* - 1) 🡪 affective well-being (*t*), (B) helicopter parenting (*t* - 1) 🡪 autonomy satisfaction (*t* - 1) 🡪 affective well-being (*t*), and (C) helicopter parenting (*t*) 🡪 autonomy satisfaction (*t*) 🡪 affective well-being (*t*). Dashed lines represent nonsignificant paths. Standardized coefficients are shown.

- - - 1. Need for relatedness.

Figure S2

*Within-Family Models Testing Relatedness Satisfaction (RS) as the Mediator at Different Temporal Orders*


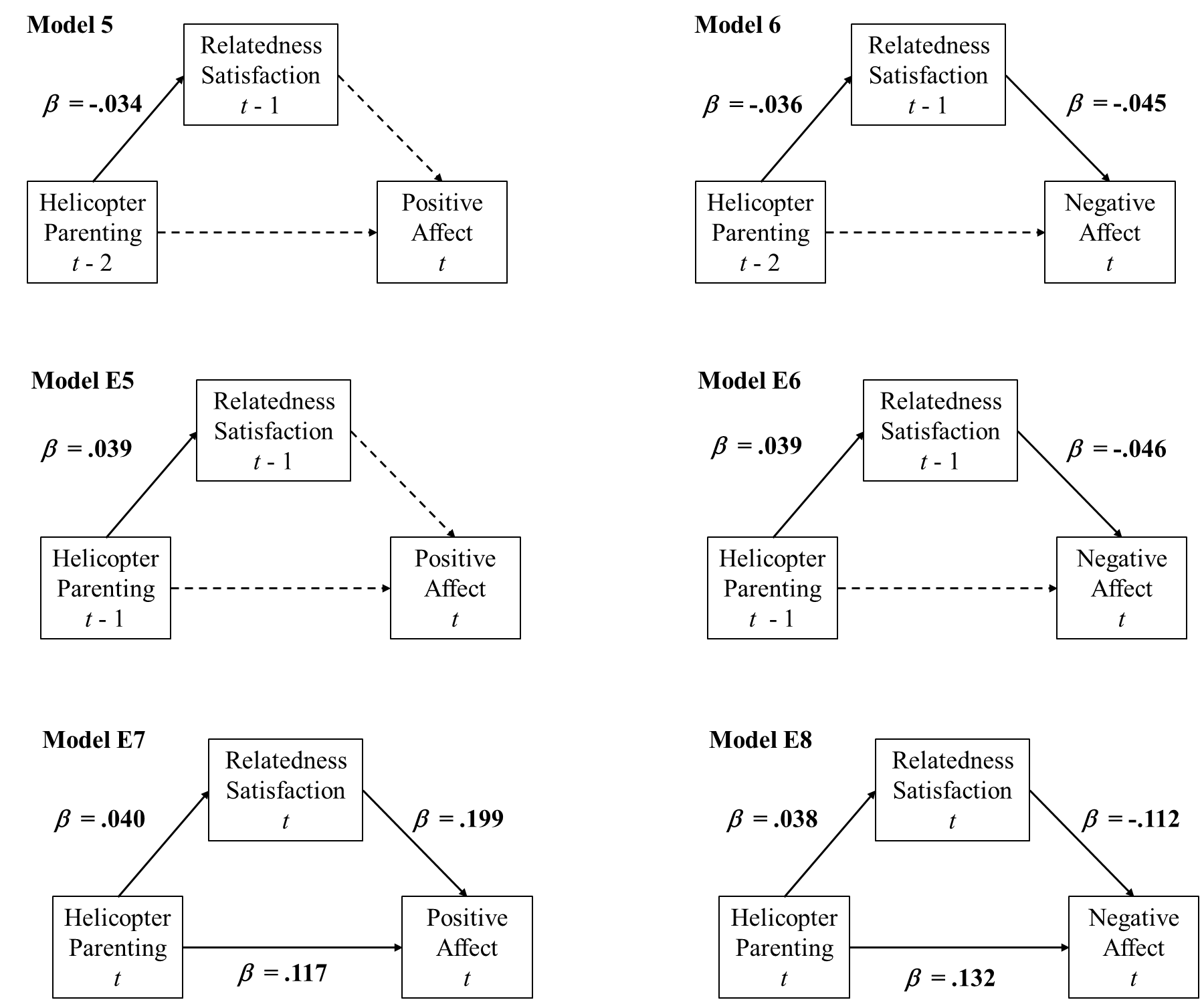


*Note.* This figure represents the mediation models: (A) helicopter parenting (*t* - 2) 🡪 relatedness satisfaction (*t* - 1) 🡪 affective well-being (*t*), (B) helicopter parenting (*t* - 1) 🡪 relatedness satisfaction (*t* - 1) 🡪 affective well-being (*t*), and (C) helicopter parenting (*t*) 🡪 relatedness satisfaction (*t*) 🡪 affective well-being (*t*). Dashed lines represent nonsignificant paths. Standardized coefficients are shown.

- - - 1. Need for competence.

Figure S3

*Within-Family Models Testing Competence Satisfaction (CS) as the Mediator at Different Temporal Orders*


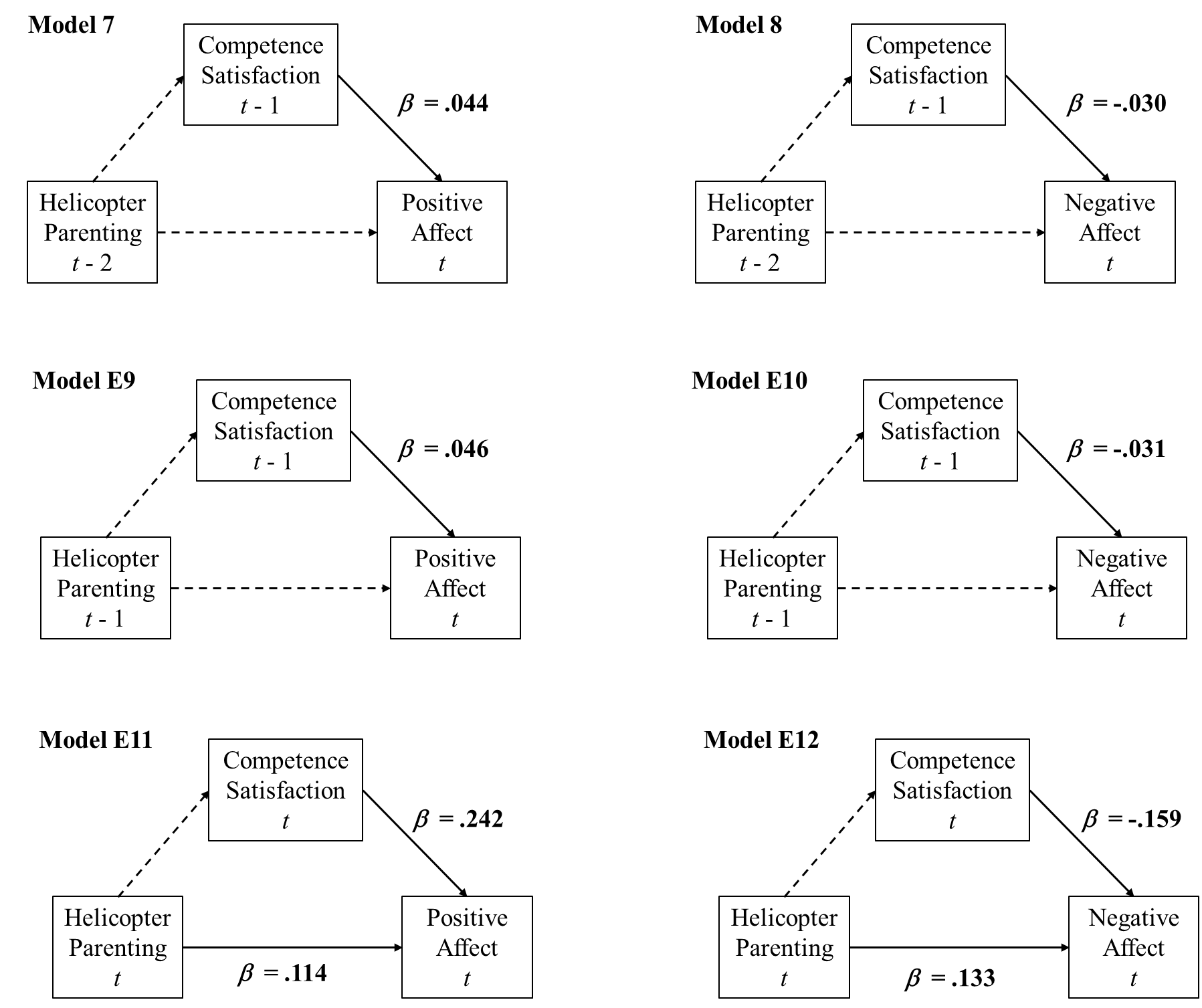


*Note.* This figure represents the mediation models: (A) helicopter parenting (*t* - 2) 🡪 competence satisfaction (*t* - 1) 🡪 affective well-being (*t*), (B) helicopter parenting (*t* - 1) 🡪 competence satisfaction (*t* - 1) 🡪 affective well-being (*t*), and (C) helicopter parenting (*t*) 🡪 competence satisfaction (*t*) 🡪 affective well-being (*t*). Dashed lines represent nonsignificant paths. Standardized coefficients are shown.

- 1. Exploratory analyses linking different helicopter parenting dimensions with different psychological needs

Since the time-lagged effects of each specific need satisfaction on youth affective well-being (both positive and negative affect) are well-established, we focused our exploratory analyses on linking each dimension of helicopter parenting to specific dimensions of need satisfaction, but not to subsequent affective well-being.

- - 1. Participants without within-person variance (ID)

Advice/Affect Management (AA, 4): 121, 162, 223, 265

Anticipatory Problem Solving (AP, 26): 15, 18, 22, 38, 51, 68, 81, 93, 107, 121, 122, 125, 126, 132, 141, 164, 176, 201, 271, 218, 223, 255, 265, 266, 314, 322

Information Seeking (IS, 4): 132, 176, 223, 265

Emphasis on Academic Performance (EA, 32): 18, 22, 36, 46, 51, 80, 92, 93, 124, 125, 128, 138, 144, 175, 176, 178, 200, 201, 202, 217, 218, 223, 235, 247, 265, 291, 314, 325, 332, 334, 343, 352

Autonomy Satisfaction (AS, 4): 75, 79, 176, 222

Relatedness Satisfaction (RS, 7): 41, 54, 75, 79, 93, 176, 244

Competence Satisfaction (CS, 11): 10, 41, 57, 76, 79, 130, 175, 176, 189, 222, 226

| Table S6 | | |  |
| --- | --- | --- | --- |
| *Sample Size for Exploratory Analyses with Different Helicopter Parenting Dimensions and Different Psychological Needs* | | | |
| Model | Average within-family | Excluded participants | *n* |
| Need for Autonomy | | |  |
| Model E13 | AA (*t* -1) 🡪 AS (*t*) | 8 | 342 |
| Model E14 | AP (*t* -1) 🡪 AS (*t*) | 29 | 321 |
| Model E15 | IS (*t* -1) 🡪 AS (*t*) | 7 | 343 |
| Model E16 | EA (*t* -1) 🡪 AS (*t*) | 35 | 315 |
| Need for Relatedness | |  |  |
| Model E17 | AA (*t* -1) 🡪 RS (*t*) | 11 | 339 |
| Model E18 | AP (*t* -1) 🡪 RS (*t*) | 31 | 319 |
| Model E19 | IS (*t* -1) 🡪 RS (*t*) | 9 | 341 |
| Model E20 | EA (*t* -1) 🡪 RS (*t*) | 38 | 312 |
| Need for competence | |  |  |
| Model E21 | AA (*t* -1) 🡪 CS (*t*) | 15 | 335 |
| Model E22 | AP (*t* -1) 🡪 CS (*t*) | 36 | 314 |
| Model E23 | IS (*t* -1) 🡪 CS (*t*) | 15 | 335 |
| Model E24 | EA (*t* -1) 🡪 CS (*t*) | 42 | 308 |

*Note*: AA = Advice/Affect Management, AP = Anticipatory Problem Solving, IS = Information Seeking, EA = Emphasis on Academic Performance, AS = Autonomy Satisfaction, RS = Relatedness Satisfaction, CS = Competence Satisfaction.

- - 1. Results For Explanatory Analyses with Different Helicopter Parenting Dimensions and Different Psychological Needs

| Table S7 | | |  |  |
| --- | --- | --- | --- | --- |
| *Results for Exploratory Analyses with Different Helicopter Parenting Dimensions and Different Psychological Needs (IN = 10,000 THIN = 2)* | | | | |
| Model | Average within-family | Est. | Est. St. | 95%CI |
| Need for Autonomy | | |  |  |
| Model E13 | Advice/Affect Management (*t* -1) 🡪 Autonomy Satisfaction (*t*) | **-0.035*** | -.036 | [-0.063, -0.005] |
| Model E14 | Anticipatory Problem Solving (*t* -1) 🡪 Autonomy Satisfaction (*t*) | **-0.039*** | -.049 | [-0.062, -0.015] |
| Model E15 | Information Seeking (*t* -1) 🡪 Autonomy Satisfaction (*t*) | 0.008 | .009 | [-0.020, 0.036] |
| Model E16 | Emphasis on Academic Performance (*t* -1) 🡪 Autonomy Satisfaction (*t*) | -0.009 | -.011 | [-0.035, 0.016] |
| Need for Relatedness | |  |  |  |
| Model E17 | Advice/Affect Management (*t* -1) 🡪 Relatedness Satisfaction (*t*) | -0.026 | -.027 | [-0.054, 0.003] |
| Model E18 | Anticipatory Problem Solving (*t* -1) 🡪 Relatedness Satisfaction (*t*) | -0.022 | -.029 | [-0.045, 0.001] |
| Model E19 | Information Seeking (*t* -1) 🡪 Relatedness Satisfaction (*t*) | -0.014 | -.014 | [-0.041, 0.013] |
| Model E20 | Emphasis on Academic Performance (*t* -1) 🡪 Relatedness Satisfaction (*t*) | **-0.032*** | -.038 | [-0.056, -0.007] |
| Need for competence | |  |  |  |
| Model E21 | Advice/Affect Management (*t* -1) 🡪 Competence Satisfaction (*t*) | -0.022 | -.022 | [-0.050, 0.006] |
| Model E22 | Anticipatory Problem Solving (*t* -1) 🡪 Competence Satisfaction (*t*) | 0.001 | .001 | [-0.022, 0.023] |
| Model E23 | Information Seeking (*t* -1) 🡪 Competence Satisfaction (*t*) | -0.023 | -.019 | [-0.051, 0.006] |
| Model E24 | Emphasis on Academic Performance (*t* -1) 🡪 Competence Satisfaction (*t*) | -0.022 | -.027 | [-0.047, 0.002] |

*Note*: Parameters whose 95% credible interval does not contain zero are shown with an asterisk. Est = unstandardized estimate. Est. St. = standardized estimate (i.e., STDYX standardization). 95%CI = Bayesian Credible Intervals of unstandardized estimate.

- 1. Exploratory analyses using mother-reported helicopter parenting

We also tested models addressing each specific need. Results indicated a consistent pattern with overall psychological need satisfaction. In this section, we only report the findings related to overall psychological need satisfaction.

- - 1. Participants without within-person variance (ID)

Mother-reported Helicopter parenting (HP): 51, 127, 135, 169, 176, 247, 291, 341

Positive affect (PA, 1): 75

Negative affect (NA, 1): 219

Autonomy Satisfaction (AS, 4): 75, 79, 176, 222

Relatedness Satisfaction (RS, 7): 41, 54, 75, 79, 93, 176, 244

Competence Satisfaction (CS, 11): 10, 41, 57, 76, 79, 130, 175, 176, 189, 222, 226

| Table S8 | | |  |
| --- | --- | --- | --- |
| *Sample Size for Exploratory Analyses with Mother-reported Helicopter Parenting* | | | |
| Model |  | Excluded participants | *n* |
| Model E25 | MHP (*t* -2) 🡪 PA (*t*) | 9 | 341 |
| Model E26 | MHP (*t* -2) 🡪 NA (*t*) | 9 | 341 |
| Model E27 | MHP (*t* -2) 🡪 AS (*t* - 1) 🡪 PA (*t*) | 11 | 339 |
| Model E28 | MHP (*t* -2) 🡪 AS (*t* - 1) 🡪 NA (*t*) | 12 | 338 |
| Model E29 | MHP (*t* -2) 🡪 RS (*t* - 1) 🡪 PA (*t*) | 14 | 336 |
| Model E30 | MHP (*t* -2) 🡪 RS (*t* - 1) 🡪 NA (*t*) | 15 | 335 |
| Model E31 | MHP (*t* -2) 🡪 CS (*t* - 1) 🡪 PA (*t*) | 19 | 331 |
| Model E32 | MHP (*t* -2) 🡪 CS (*t* - 1) 🡪 NA (*t*) | 19 | 331 |

Note. MHP = Mother-reported helicopter parenting. AS = Autonomy Satisfaction, RS = Relatedness Satisfaction, CS = Competence Satisfaction

- - 1. Results For Explanatory Analyses using Mother-reported Helicopter Parenting

| Table S9 |  | | |  |  | | |
| --- | --- | --- | --- | --- | --- | --- | --- |
| *Results for Exploratory Analyses with Mother-reported Helicopter Parenting (IN = 10,000 THIN = 2)* | | | | | | | |
|  | Positive Affect (PA) | | |  | Negative Affect (NA) | | |
| Average within-family | Est. | Est. St. | 95%CI |  | Est. | Est. St. | 95%CI |
| **Total Effects (Model E25-E26)** |  |  |  |  |  |  |  |
| Helicopter parenting (*t* - 2) 🡪 Affect (*t)* | 0.004 | .005 | [-.015, .022] |  | **0.017*** | .029 | [0.002, 0.033] |
| **Autonomy Satisfaction (Model E27-E28)** |  |  |  |  |  |  |  |
| Helicopter parenting (*t* - 1) 🡪 Autonomy satisfaction (*t*) | 0.004 | .007 | [-0.018, 0.026] |  | 0.002 | .003 | [-0.016, 0.021] |
| Autonomy satisfaction (*t* - 1) 🡪 Affect (*t*) | **0.023*** | .030 | [0.000, 0.048] |  | **-0.025*** | -.035 | [-0.046, -0.004] |
| Helicopter parenting (*t* - 2) 🡪 Autonomy satisfaction (*t* - 1) 🡪Affect (*t)* | 0.000 | - | [0.000, 0.001] |  | 0.000 | - | [-0.001, 0.000] |
| **Relatedness Satisfaction (Model E29-E30)** |  |  |  |  |  |  |  |
| Helicopter parenting (*t* - 1) 🡪 Relatedness satisfaction (*t*) | -0.011 | -.014 | [-0.032, 0.011] |  | -0.010 | -.013 | [-0.032, 0.012] |
| Relatedness satisfaction (*t* - 1) 🡪 Affect (*t*) | **0.025*** | .032 | [0.001, 0.049] |  | **-0.037*** | -.051 | [-0.060, -0.015] |
| Helicopter parenting (*t* - 2) 🡪 Relatedness satisfaction (*t* - 1) 🡪Affect (*t)* | 0.000 | - | [-0.001, 0.000] |  | 0.000 | - | [0.000, 0.001] |
| **Competence Satisfaction (Model E31-E32)** |  |  |  |  |  |  |  |
| Helicopter parenting (*t* - 1) 🡪 Competence satisfaction (*t*) | 0.002 | .002 | [-0.022, 0.025] |  | 0.001 | .002 | [-0.022, 0.024] |
| Competence satisfaction (*t* - 1) 🡪 Affect (*t*) | **0.033*** | .043 | [0.009, 0.057] |  | -0.020 | -.055 | [-0.041, 0.000] |
| Helicopter parenting (*t* - 2) 🡪 Competence satisfaction (*t* - 1) 🡪Affect (*t)* | 0.000 | - | [-0.001, 0.001] |  | 0.000 | - | [-0.001, 0.001] |

*Note*: Parameters whose 95% credible interval does not contain zero are shown with an asterisk. Est = unstandardized estimate. Est. St. = standardized estimate (i.e., STDYX standardization). 95%CI = Bayesian Credible Intervals of unstandardized estimate.
